# Assessment of Risk Factors Leading to Amputation Among Diabetic Septic Foot Patients in Khartoum, Sudan

**DOI:** 10.7759/cureus.75517

**Published:** 2024-12-11

**Authors:** Mohamed Elmubark, Lamis Fahal, Faris Ali, Hosam Nasr, Abdulrahman Mohamed, Kenechukwu Igbokwe

**Affiliations:** 1 Faculty of Medicine, The National Ribat University, Khartoum, SDN; 2 Orthopaedics, The Dudley Group National Health Services (NHS) Foundation Trust, Birmingham, GBR; 3 Faculty of Medicine, University of Medical Sciences and Technology, Khartoum, SDN; 4 Trauma and Orthopaedics, Gateshead Health National Health Services (NHS) Foundation Trust, Gateshead, GBR

**Keywords:** amputation, diabetes, hba1c, neuropathy, orthopaedics, septic foot, trauma

## Abstract

Introduction

Diabetes is a rapidly growing global health concern, with the World Health Organization (WHO) estimating that 300 million adults will have diabetes by 2025. This chronic condition is associated with complications, including nephropathy, retinopathy, neuropathy, cardiovascular disease, and diabetic foot ulcers (DFUs), which can lead to amputation. Diabetic septic foot (DSF), a severe form of diabetic foot disease, is defined by the WHO as the presence of infection, ulceration, or tissue destruction in the lower limb, often accompanied by neurological abnormalities, peripheral vascular disease, and metabolic complications of diabetes. In Sudan, the incidence of lower limb amputations due to DSF is increasing, with various healthcare centers employing different management strategies, making it challenging to identify which factors are most strongly linked to the highest rates of amputations. This study aims to identify the key risk factors contributing to amputations in patients with diabetic foot syndrome (DFS) in Khartoum, Sudan. Specifically, it seeks to assess the common risk factors for amputation in diabetic septic foot patients at hospital wards and dressing centers in Khartoum State, Sudan. Objectives include identifying risk factors associated with amputations, determining the types and frequency of amputations, and evaluating diabetes control and foot self-care practices.

Methods

This was a descriptive cross-sectional study that was conducted anonymously between 01/11/2017 and 08/11/2017 in various hospital wards and dressing centers in Khartoum, Sudan, on 46 diabetic septic foot patients. Data was collected using an interview questionnaire and checklist administered to the attending patients. Quantitative data was analyzed using IBM Corp. Released 2011. IBM SPSS Statistics for Windows, Version 20.0. Armonk, NY: IBM Corp. This paper aims to explore the pathophysiology, risk factors, and clinical management of diabetic foot complications, with a focus on preventing the devastating outcome of amputation.

Results

The findings revealed that the most significant risk factors for amputation included a raised HbA1c level (74%), male gender (78%), age over 50 years (96%), and a history of preceding non-healing ulcers (93.5%). Other factors, such as smoking, diabetes-related comorbidities, and the type of diabetes management, showed no significant association with amputation. Normal saline and iodine were the most commonly used wound care solutions (52%). Regarding foot care habits, the most frequently practiced measures among patients included wearing special diabetic shoes (63%), avoiding walking barefoot (63%), and refraining from smoking (59%).

Conclusions

This study identified key risk factors for amputations in diabetic septic foot (DSF) patients, including elevated HbA1C levels, male gender, age over 50, and a history of unhealing ulcers, with poor foot care practices contributing to higher amputation rates. The findings highlight the importance of glycemic control, foot hygiene, and patient education in preventing amputations. Additionally, the study underscores the need for comprehensive management strategies that address both metabolic control and foot care, particularly in resource-limited settings. These insights can guide local healthcare policies focused on prevention, early intervention, and better resource allocation to reduce diabetes-related complications and improve patient outcomes.

## Introduction

Diabetes is a rapidly growing global health concern, with the World Health Organization (WHO) estimating that 300 million adults will have diabetes by 2025 [1]. This chronic condition is associated with numerous complications, including nephropathy, retinopathy, neuropathy, cardiovascular disease, and diabetic foot ulcers (DFUs), all of which are expected to increase as the number of diabetes cases rises [2]. Among these, DFUs are particularly problematic, often overlooked in their early stages, and if left untreated, can lead to amputation [3]. The impact of diabetic foot ulcers and amputations extends beyond patient health, creating significant socioeconomic challenges, reducing quality of life, and placing a heavy economic burden on individuals and society [4].

Diabetic septic foot (DSF), a severe form of diabetic foot disease, is defined by the WHO as the presence of infection, ulceration, or tissue destruction in the lower limb, often accompanied by neurological abnormalities, peripheral vascular disease, and metabolic complications of diabetes [5]. DSF is a leading cause of non-traumatic lower extremity amputation (LEA), with foot ulcers being the most common precursor [6]. The prevalence of foot ulcers among diabetic patients ranges from 4% to 10%, with a lifetime incidence of up to 25% [7].

The pathophysiology of diabetic foot disease begins with a small ulcer or injury that, if not properly managed, may progress to a non-healing wound, ultimately resulting in LEA. Approximately 82% of LEAs are performed on diabetic patients, most of whom developed foot ulcers [8-10]. Contributing factors such as peripheral neuropathy, vascular insufficiency, and compromised immunity heighten the risk of infections and poor wound healing [11]. In regions like Sudan, poor diabetes control, inadequate foot care, and cultural dietary habits exacerbate these risks.

Amputation, the surgical removal of a limb or part of it, is often a last resort when the affected tissue becomes irreparable or threatens systemic health [12]. The primary indications for amputation include "dead" tissue with no blood supply, "deadly" conditions such as wet gangrene or malignancy, and "dead loss," where the limb is nonfunctional or impeding quality of life [13]. Amputations are classified into minor (e.g., digit or partial foot) and major (e.g., below-knee, above-knee, or hip disarticulation) types, each with distinct clinical indications [12].

Early recognition and management of diabetic foot ulcers and their risk factors are crucial for preventing amputations. This paper aims to explore the pathophysiology, risk factors, and clinical management strategies for diabetic foot complications, with a focus on preventing the devastating outcomes of amputations.

In Sudan, the incidence of lower limb amputations due to DSF is increasing, with various healthcare centers employing different management strategies, making it challenging to identify which factors are most strongly linked to the highest rates of amputations [6]. Many patients are from low socioeconomic backgrounds and may have limited access to education on diabetes care and foot health, exacerbating the risk [7]. This highlights the urgent need for improved health education and specialized care in managing diabetic foot complications.

This study aims to identify the key risk factors contributing to amputations in patients with diabetic foot syndrome (DFS) in Khartoum, Sudan. Specifically, it seeks to assess the common risk factors for amputation in diabetic septic foot patients at hospital wards and dressing centers in Khartoum State, Sudan. Objectives include identifying risk factors associated with amputations, determining the types and frequency of amputations, and evaluating diabetes control and foot self-care practices. 

## Materials and methods

This study was conducted between 01/11/2017 and 08/11/2017 in various hospital wards and dressing centers in Khartoum State, Sudan, on 46 diabetic septic foot (DSF) patients who were undergoing treatment or had undergone amputation. The study aimed to identify common risk factors leading to amputations in this patient group and assess related clinical outcomes. Data were collected through structured interviews and medical record reviews, with a focus on demographic information, diabetes control, foot care practices, and amputation details. The study was carried out in accordance with the Declaration of Helsinki, with ethical approval from the National Ribat University institutional review board (IRB: NRU-MED-CMD-1643892017) and the Khartoum State Ministry of Health (MOH). Informed consent was obtained from all participants.

Study design and setting

A cross-sectional descriptive study design was employed, involving multiple healthcare centers that treat DSF patients in Khartoum State. These included specialized diabetes care hospitals and dressing centers, where patients with diabetic foot ulcers or amputations were regularly treated. The selected centers were chosen for their role in managing diabetic foot complications and amputation cases.

Participants

The study targeted diabetic septic foot patients attending the selected hospitals and dressing centers. The total number of participants involved in this study was 46 patients. Patients inclusion criteria included patients diagnosed with diabetes mellitus who either had undergone or were at risk of amputation due to diabetic foot complications. Patients were interviewed following ethical approval, and written consent was obtained prior to participation.

Data collection

The data was gathered anonymously between 01/11/2017 and 08/11/2017 using a combination of interview questionnaires and patient medical records. The interview questionnaires were designed to assess risk factors such as diabetes control (HbA1c levels), foot self-care practices, smoking habits, and comorbidities like hypertension and obesity. The medical records provided detailed information on the type of amputation, the number of amputations performed, and the progression of diabetic foot disease. 

Variables assessed

Key Variables Included

Risk factors for amputation: The study examined factors such as poorly controlled diabetes, peripheral neuropathy, vascular insufficiency, smoking, and inadequate foot care.

Types and number of amputations: The study categorized amputations into minor (e.g., toe or partial foot amputation) and major (e.g., below-knee or above-knee amputation) types, documenting the frequency of each.

Diabetes control and foot care: Participants were asked about their diabetes management, including HbA1c levels, use of medications, and adherence to foot care practices such as wearing diabetic footwear and regular foot inspections.

Demographics: The relationship between age, sex, and the occurrence of amputations was also explored.

Patient awareness: The study assessed patients' knowledge of the importance of preventing diabetic foot complications and their awareness of proper foot care techniques.

Inclusion criteria

The inclusion criteria for this study comprised diabetic patients who had previously undergone amputations related to diabetic foot syndrome. In addition, the criteria included diabetic patients with severe ulcers and those awaiting amputation as a result of diabetic foot syndrome attending wards of our selected hospitals and dressing centers during the specified data collection period. By focusing on this specific patient population, the study aimed to gather relevant data on the experiences and outcomes of individuals affected by diabetes and its complications, ensuring that the findings would be pertinent to addressing the needs of these patients effectively.

Exclusion criteria

Diabetic patients who had no signs of diabetic septic foot or foot ulcers with a risk of amputation were excluded from our study, as were non-diabetic patients who had undergone lower leg or foot amputations and were attending our selected dressing centers and specified hospitals at the time of data collection. Additionally, diabetic patients with amputations, foot ulcers, or signs of diabetic septic foot with the threat of amputation who were not present on the designated data collection days were also excluded from the study to ensure the accuracy and reliability of the data. Diabetic septic foot patients with severe ulcers who had undergone amputations and who were severely unwell or obtunded at the time of data collection were not included, as their condition affected their ability to participate in the study. 

Data analysis

Data were analyzed using IBM Corp. Released 2011. IBM SPSS Statistics for Windows, Version 20.0. Armonk, NY: IBM Corp. to identify trends and correlations. Descriptive statistics were used to summarize the prevalence of risk factors and types of amputations, while inferential statistics helped explore the relationships between demographic factors, diabetes control, and amputation outcomes.

By employing these methods, the study aimed to provide a comprehensive understanding of the factors contributing to amputations in DSF patients in Khartoum, with the ultimate goal of improving prevention strategies and patient outcomes.

## Results

Demographics

The majority of amputations occurred in individuals over the age of 50 (44 patients) (Table 1). Specifically, those aged over 60 years accounted for 24 patients who had undergone amputation, while there were 20 patients aged 50-60 years who underwent amputation. Only two patients under the age of 30 underwent amputation.

**Table 1:** Number of amputations by age amongst diabetic septic foot patients

Age Group	Number of Amputations
Over 60 years	24
50-60 years	20
Under 30 years	2
Total	46

Out of a total of 46 amputees, the majority were male, accounting for 36 individuals (Table 2). In contrast, female patients comprised a smaller portion, with only 10 individuals of the total diabetic septic foot amputee population. This data indicates a significant gender disparity in the incidence of amputations, with male patients being more likely to undergo the procedure than female patients.

**Table 2:** Number of amputees by gender

Gender	Number of Amputees
Male	36
Female	10
Total	46

Among the total of 46 amputees, the longest duration category, 16-20 years, had the highest number of amputations, with 12 individuals (Table 3). The next most significant group was those who had lived with diabetes for 21 years or more, accounting for 10 amputees. Additionally, nine amputees fell into the 6-10 years category, while eight individuals had diabetes for 11-15 years. Only seven amputees had been diagnosed with diabetes for five years or less. 

**Table 3:** Duration of diabetes in patients who underwent amputation

Duration of Diabetes	Number of Amputations
1-5 years	7
6-10 years	9
11-15 years	8
16-20 years	12
21+ years	10
Total	46

**Figure 1:**
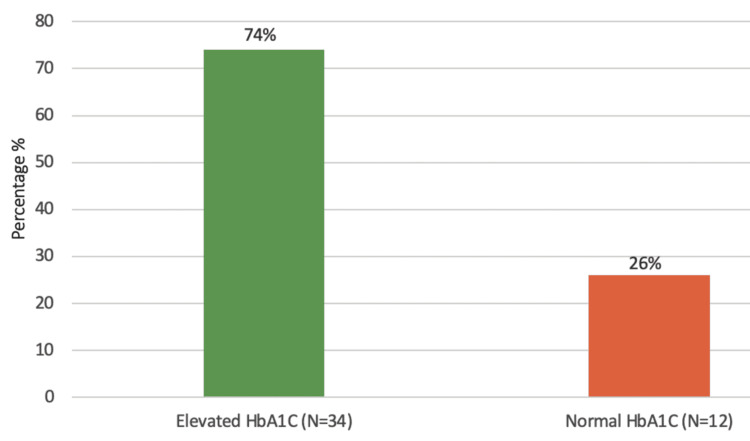
HbA1C levels amongst diabetic septic foot patients undergoing amputation

Among the patients requiring amputation, 34 individuals, representing 74%, exhibited elevated HbA1C levels. In contrast, 12 patients, accounting for 26%, had normal HbA1C levels (Figure 1). This indicates a significant association between elevated HbA1C levels and the necessity for amputation among this patient population.

**Figure 2:**
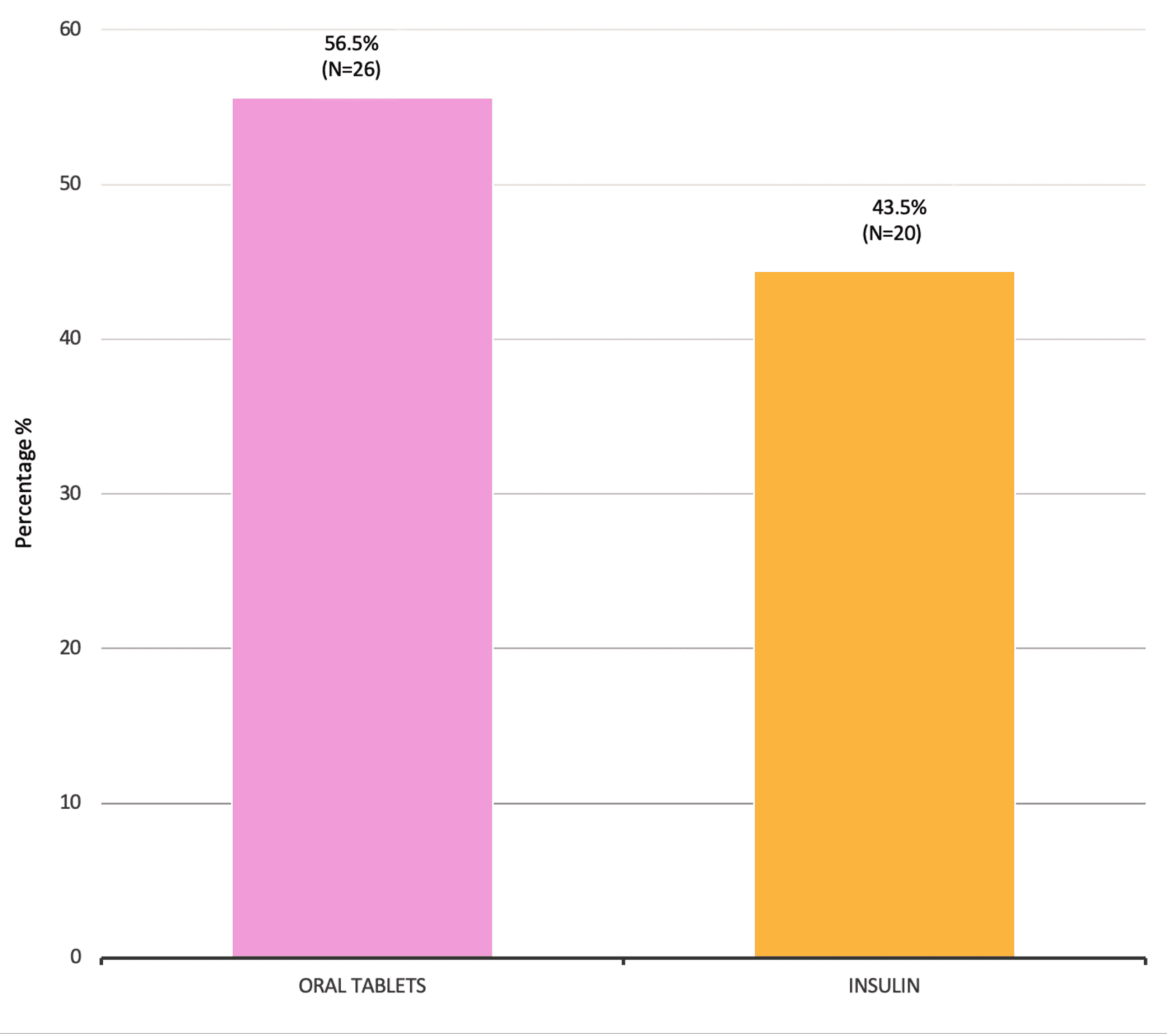
Diabetic control by medication amongst diabetic septic foot patients

In the context of diabetes management among the patient population, the data revealed that 56% (N=26) of patients took oral tablets to control their diabetes, while 44.4% (N=20) used insulin as their treatment method (Figure 2). This finding suggests that both treatment approaches may carry similar risks regarding the necessity for amputations, highlighting the importance of effective diabetes management regardless of the treatment method chosen.

**Figure 3:**
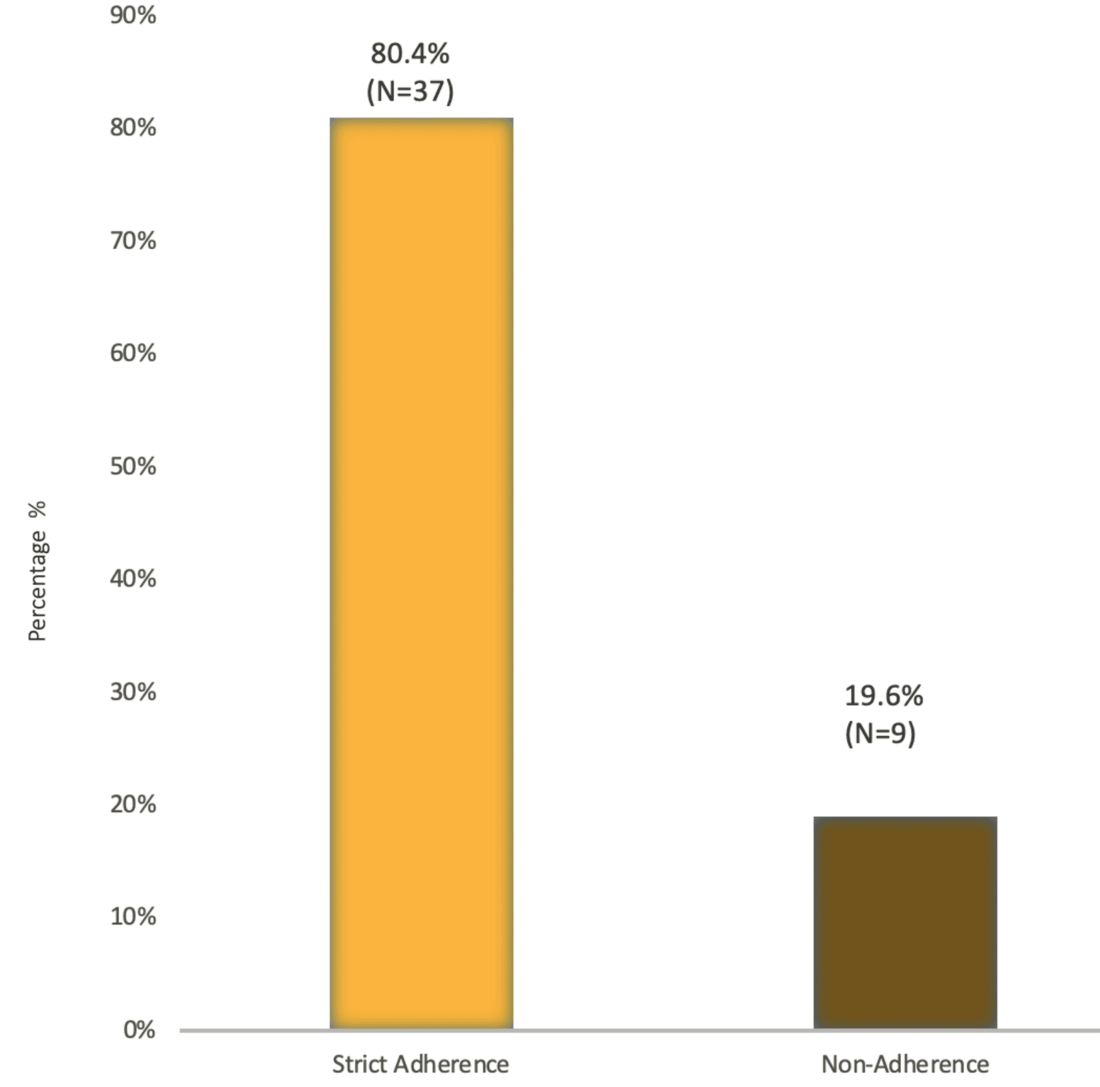
Adherence to regular medication intake amongst diabetic septic foot patients

Most patients (80.4%) adhered strictly to their medication regimen (Figure 3), yet still required amputations, suggesting that medication adherence alone was not sufficient to prevent DSF-related amputations.

**Figure 4:**
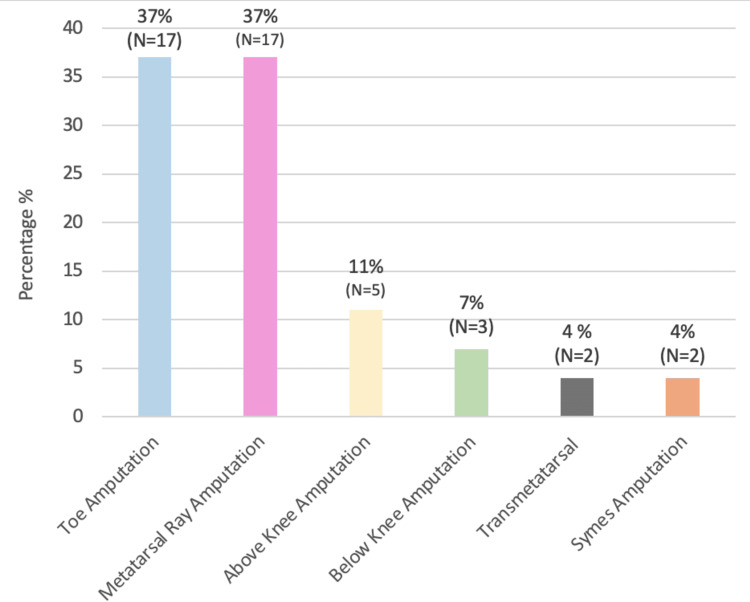
Types of amputation amongst diabetic septic foot patients

The most common types of amputations were toe amputations and metatarsal ray amputations, both of which accounted for 37% of all amputations in the study population (Figure 4). Transmetatarsal and Symes accounted for 4% each, and above-knee and below-knee amputations accounted for 11% and 7%, respectively.

**Figure 5:**
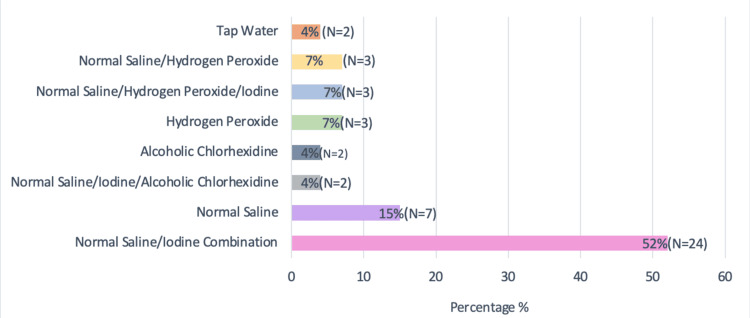
Types of dressing solutions used by diabetic septic foot patients

The most commonly used wound-cleaning solution among patients was a combination of normal saline and iodine, accounting for 52% of treatments. This preference may be due to the widespread availability and affordability of these solutions in Sudanese hospitals and dressing centers (Figure 5).

**Figure 6:**
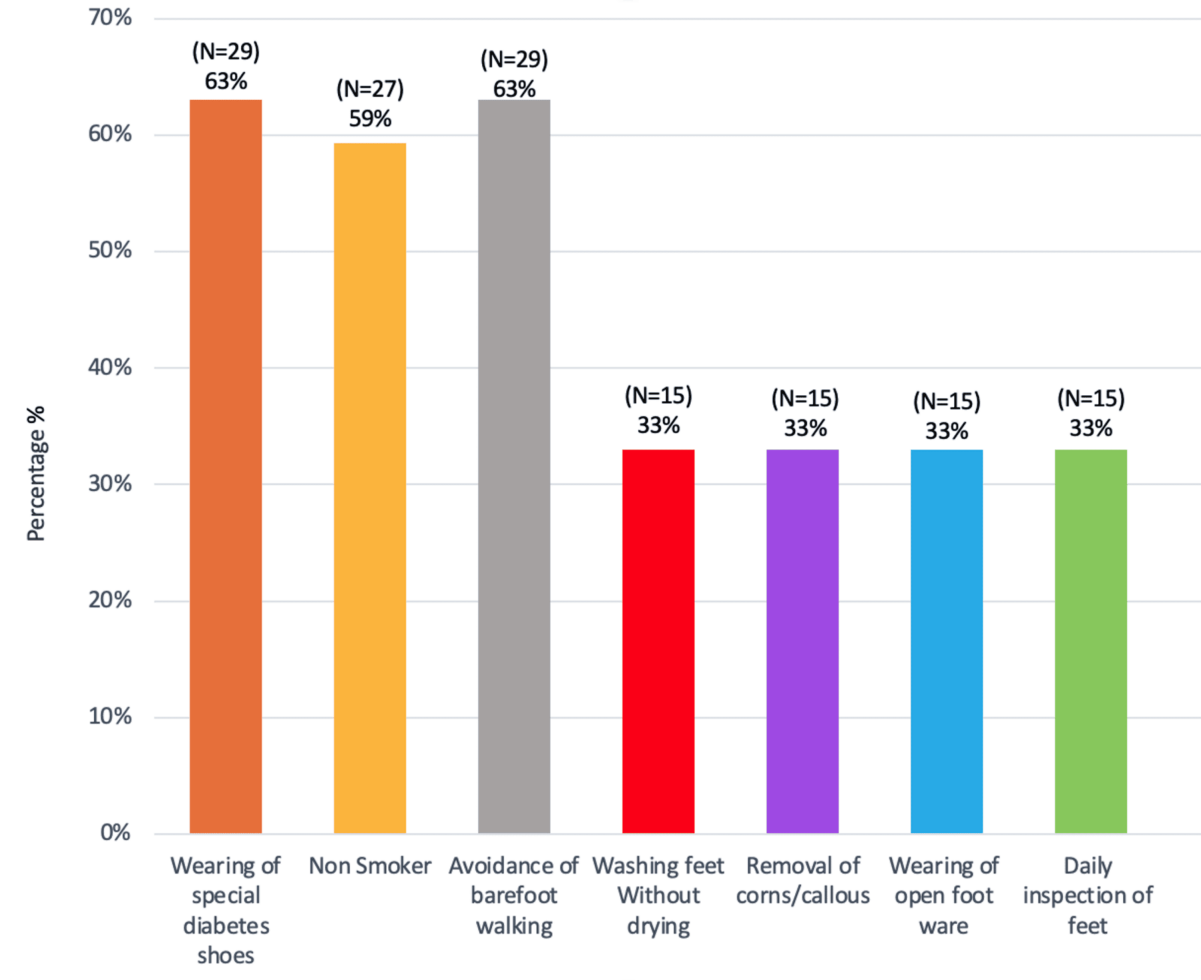
Practiced foot care habits amongst diabetic septic foot patients

Foot care habits varied among DSF patients. The most commonly practiced habits included wearing special diabetic shoes (63%), avoiding walking barefoot (63%), and avoiding smoking (59%). However, other critical habits, such as drying feet after washing, avoidance of wearing open footwear, and daily foot inspections, were only adhered to by approximately 33% of patients (Figure 6). This lack of proper foot care may have contributed to the high rate of amputations.

**Figure 7:**
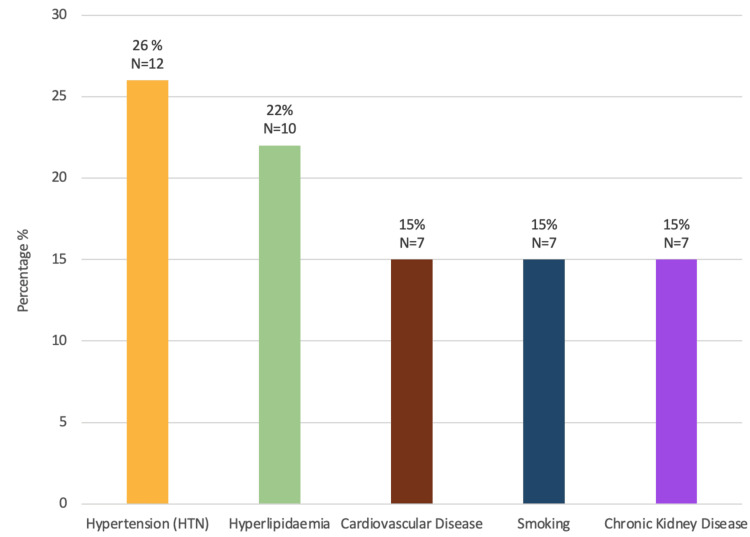
Co-morbidities amongst diabetic septic foot patients

As seen in Figure 7, hypertension (26%) was the most common co-morbidity found in DSF patients, followed by hyperlipidemia (22%). However, the relatively low incidence of co-morbidities suggests that these factors played a minimal role in the progression to amputation in our cohort.

**Figure 8:**
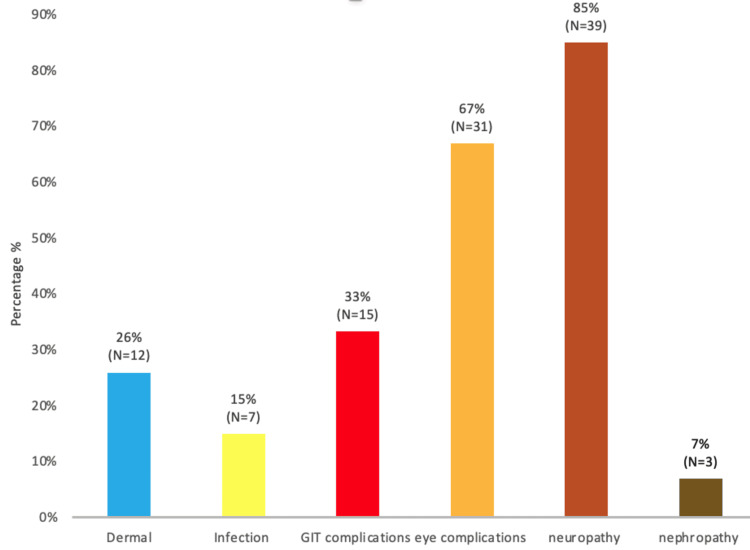
Complications amongst diabetic septic foot amputees

The single most common complication experienced by DSF patients was neuropathy (85%), followed secondly by eye complications (Figure 8).

**Figure 9:**
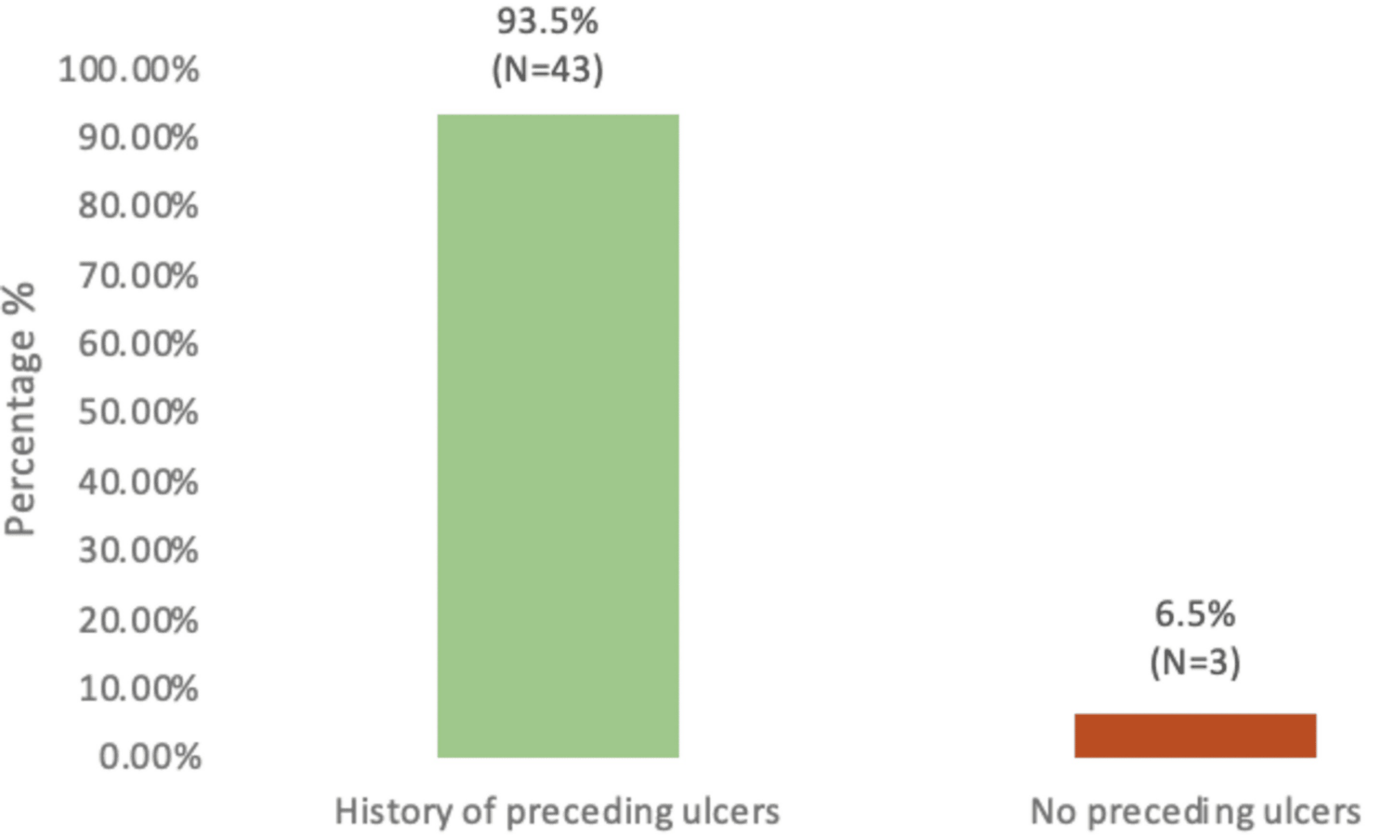
History of preceding non-healing ulcers amongst diabetic septic foot patients

A key finding in our research was that 93.5% of DSF patients had a history of preceding non-healing ulcers that had been present for more than two weeks (Figure 9).

## Discussion

The results of our study highlight several important factors associated with amputations in diabetic septic foot (DSF) patients in Khartoum. In a previous study involving 81 patients admitted with diabetic septic foot at Wad-Medni Teaching Hospital, Medni, Sudan, between 2008 and 2009 [14], the mean age of DSF patients was reported as 55.6 years, with a male-to-female ratio of 2:1. This age distribution aligns with our findings, which show that the mean age in which amputation occurs is > 50 years, with a total of 96% of amputations occurring within this age group. This reinforces the notion that age is a significant factor in the occurrence of diabetic foot complications leading to amputation. The prevalence of amputations in our study showed a higher amputation rate in males (78%) compared to females (22%). Similarly, the study at Wad Medni and other studies reviewed as part of our discussion at the Military Hospital Khartoum [15] and Omdurman Teaching Hospital [16] confirmed a similar trend with a male predominance among DSF cases, suggesting a consistent pattern across similar studies regarding gender differences in the prevalence of DSF and related amputations. This suggests that male diabetic patients are at a higher risk for complications that lead to amputations, likely due to a combination of biological, socio-cultural, and behavioral factors. Understanding this gender difference can help in targeting preventive strategies and health interventions, particularly for males, who might benefit from more focused education on foot care and early intervention to reduce the risk of severe outcomes like amputations. Neuropathy was the most common complication amongst amputees within our cohort (85%), followed by eye complications (67%), in keeping with the findings of the Wad Medni study in which neuropathy was highlighted as a significant complication related to amputations, establishing a consistent correlation between neuropathy and poor outcomes in patients with DSF. 

We identified a number of risk factors in diabetic amputees, including comorbidities such as HTN (26%) and hyperlipidemia (22%), as well as high HbA1C level (74%) as a significant risk factor for lower extremity amputations (LEA) amongst our patients. Balla et al. conducted a case-control study among diabetic patients attending the diabetic clinic in the Military Hospital, Khartoum, Sudan, during May-June 2012, which included 30 DSF cases and 30 controls [15]. This study identified foot deformity (9.3%), absence of arterial pulse (9%), and peripheral neuropathy (5%) as strong associations with amputations. Together, these studies emphasize the multifaceted nature of diabetic septic foot complications and the critical need for comprehensive management strategies that address both metabolic control and the prevention of foot-related complications. This approach is vital to reducing the incidence of amputations and improving overall patient outcomes in diabetic populations. The mean age of participants in the military hospital study was 55.60 ± 11.9 years, indicating a similar age demographic among patients affected by DSF to those included in our study. A key finding in our research was that 93.5% of DSF patients had a history of preceding non-healing foot ulcers that had been present for more than two weeks. This finding was similar to the study conducted at the military hospital of Khartoum, which found that 70% of cases with foot ulcer patients presented to the hospital after 1-2 weeks of noticing the ulcer, indicating delays in seeking treatment. This delay is a significant concern, as it suggests a lack of awareness or understanding of the severity of foot ulcers among patients, potentially leading to worsened outcomes. Our study showed that 56.5% of patients used oral medications to control diabetes while 43.5% used insulin and that a high adherence rate to medication (80.4%) did not prevent amputations. The military hospital study highlighted that 20% of patients used insulin and that the majority of patients (80%) used oral hypoglycemic agents, similar to our findings regarding treatment methods. Although this indicates a common trend in diabetic management, it suggests that control methods are not sufficiently effective in preventing DSF and subsequent amputations. When we assessed practiced habits amongst diabetic septic foot patients, 63% of patients wore special diabetic shoes and avoided walking barefoot, indicating some awareness of foot care. In the study conducted by Balla et al., only 7.9% of diabetic patients wore special shoes and 4.6% undertook daily self-examination of the feet. This suggests that while some protective behaviors exist amongst diabetic septic foot patients in Sudan, they are not universally practiced, indicating that there is room for improvement in education and promotion of self-care among patients.

Adam et al. conducted a retrospective study on 208 patients with DSF admitted to Omdurman Teaching Hospital, Sudan, between June 2006 and May 2007 [16] to audit the management of diabetic septic foot lesions in Omdurman Teaching Hospital, using the Wagner classification. This study showed outcomes recorded for managing patients, which showed that 14.4% of patients were cured, 78.8% had improved, and 6.7% of patients had died. Although we focused on the characteristics of amputees, noting the incidence of complications like neuropathy and risk factors such as previous ulcers that lead to amputation, without looking at specific outcomes, the significant improvement rate in the Omdurman study indicates that many patients can recover when modifiable risk factors amongst diabetic septic foot patients are addressed and controlled. The Omdurman study emphasized the importance of early presentation and preventative measures for the management of diabetic foot lesions. As seen in our study, the importance of foot care practices and neglect of essential behaviors like daily foot inspection contribute to significant complications such as amputation. Both studies therefore agree on the critical need for preventive education in managing DSF, indicating that better foot care practices could significantly impact outcomes.

Study limitations

Several challenges were encountered during the study. The time available for data collection was limited, which may have affected the depth of the research. Despite obtaining the necessary governmental approvals, some centers were initially hesitant to cooperate, leading to delays in the research process. Additionally, locating and recruiting the study population proved to be difficult, as identifying diabetic septic foot patients within the specified timeframe was challenging. These factors may have impacted the study's scope and its overall findings. Furthermore, the study's reliance on patients from the Khartoum area alone may limit the generalizability of the findings to other regions in Sudan or neighboring countries, where healthcare infrastructure and population demographics could differ. Additionally, the retrospective nature of the data collection may introduce biases, such as the potential underreporting of certain risk factors, which could affect the accuracy of our findings.

## Conclusions

This study identified key risk factors for amputations in diabetic septic foot (DSF) patients, including elevated HbA1C levels, male gender, age over 50, and a history of unhealing ulcers, with poor foot care practices contributing to higher amputation rates. The findings highlight the importance of glycemic control, foot hygiene, and patient education in preventing amputations. Additionally, the study underscores the need for comprehensive management strategies that address both metabolic control and foot care, particularly in resource-limited settings. These insights can guide local healthcare policies focused on prevention, early intervention, and better resource allocation to reduce diabetes-related complications and improve patient outcomes. 
